# Adenylate Cyclase Toxin promotes bacterial internalisation into non phagocytic cells

**DOI:** 10.1038/srep13774

**Published:** 2015-09-08

**Authors:** César Martín, Asier Etxaniz, Kepa B. Uribe, Aitor Etxebarria, David González-Bullón, Jon Arlucea, Félix M. Goñi, Juan Aréchaga, Helena Ostolaza

**Affiliations:** 1 Departamento de Bioquímica y Biología Molecular and Unidad de Biofísica (CSIC, UPV/EHU), Universidad del País Vasco, Aptdo. 644, 48080 Bilbao, Spain.; 2 Departamento de Biología Celular, Facultad de Medicina, Universidad del País Vasco, Aptdo. 644, 48080 Bilbao, Spain

## Abstract

*Bordetella pertussis* causes whooping cough, a respiratory infectious disease that is the fifth largest cause of vaccine-preventable death in infants. Though historically considered an extracellular pathogen, this bacterium has been detected both *in vitro* and *in vivo* inside phagocytic and non-phagocytic cells. However the precise mechanism used by *B. pertussis* for cell entry, or the putative bacterial factors involved, are not fully elucidated. Here we find that adenylate cyclase toxin (ACT), one of the important toxins of *B. pertussis*, is sufficient to promote bacterial internalisation into non-phagocytic cells. After characterization of the entry route we show that uptake of “toxin-coated bacteria” proceeds *via* a clathrin-independent, caveolae-dependent entry pathway, allowing the internalised bacteria to survive within the cells. Intracellular bacteria were found inside non-acidic endosomes with high sphingomyelin and cholesterol content, or “free” in the cytosol of the invaded cells, suggesting that the ACT-induced bacterial uptake may not proceed through formation of late endolysosomes. Activation of Tyr kinases and toxin-induced Ca^2+^-influx are essential for the entry process. We hypothesize that *B. pertussis* might use ACT to activate the endocytic machinery of non-phagocytic cells and gain entry into these cells, in this way evading the host immune system.

Whooping cough, caused by the Gram-negative bacterium *Bordetella pertussis*, remains an important public health concern, as highlighted by the increasing number of disease outbreaks worldwide and the rise in infant mortality despite widespread (>90%) vaccination programs^1^,^2^. *B. pertussis* was regarded as a noninvasive pathogen that caused disease through the action of various potent virulence factors^3^,^4^. The successful persistence of this pathogen has been mainly attributed to its ability to interfere with various aspects of the immune system, from the inhibition of complement- and phagocyte-mediated killing to the suppression of T- and B-cell responses^3^,^5^. However, a number of reports have noted that virulent *B. pertussis* may exist and even replicate inside phagocytic and non-phagocytic cells, both *in vitro* and *in vivo*^6^,^7^,^8^,^9^,^10^,^11^, suggesting that *B. pertussis* may have developed mechanisms of cell invasion to evade an active host immune response. The precise mechanism used by *B. pertussis* for cell entry, or the putative bacterial factors involved in invasion are not yet fully understood.

*B. pertussis* expresses an ample repertoire of virulence factors: adhesins such as filamentous hemagglutinin (FHA), fimbriae, and pertactin^12^,^13^, as well as various toxins including tracheal cytotoxin, dermonecrotic toxin, pertussis toxin, and adenylate cyclase toxin (ACT; also known as CyaA)^12^,^13^. Whether these adhesins and toxins contribute to *B. pertussis* invasivity is not still fully clear, as contradictory results have been described to date. While some authors reported that adhesins such as FHA or pertactin, and toxins such as pertussis toxin induced *B. pertussis* invasion in HeLa 229 cells, A549 cells (alveolar basal epithelial cells) or Hep-2 cells (epidermoid carcinoma cells)^7^,^14^,^15^, others reported that pertussis toxin or FHA were not involved in the invasion process^6^,^10^,^16^. The involvement of ACT in *B. pertussis* invasion also remains obscure. Early reports had suggested that ACT was not involved in invasion, as mutant *B. pertussis* strains lacking ACT were capable of invading HeLa 229 cells, others have suggested that ACT inhibits bacterial invasion in human tracheal epithelial cells (HTE) and in HeLa cells^6^,^7^ and other group did not found evidence for a significant inhibitory effect of ACT in the entry of *B. pertussis* into A549 cells^10^.

ACT is a 200 kDa protein with two functional domains: a N-terminal adenylate cyclase enzymatic domain (AC domain) and a C-terminal hemolysin domain (Hly domain)^17^ with characteristic glycine/aspartate-rich Ca^2+^-binding repeats typically present in the members of the RTX (Repeats in Toxin) family of proteins, including ACT^17^,^18^,^19^,^20^. The hemolysin domain mediates binding to CD11b/CD18, the ACT receptor^21^,^22^ and direct translocation of the AC catalytic domain into the cell cytosol^17^. Upon activation by cellular calmodulin, this translocated domain catalyzes conversion of ATP to cAMP^17^,^23^. ACT exerts, via cAMP generation, immunosuppressive and immunoregulatory effects on both the innate and adaptive immune systems^24^,^25^,^26^,^27^,^28^,^29^. Though CD11b/CD18 expressing-myeloid cells are the most susceptible ACT targets, non-immune cells, such as epithelial cells, are also susceptible to toxin activity, though at higher toxin concentrations^30^. Although it has classically been accepted that the unregulated increase in intracellular cAMP levels underlies ACT’s cytotoxic activity, this toxin exhibits other functions, not all of which cause cell death, i.e. inhibition of cell proliferation^31^.

Recently, our group has reported that purified ACT is internalised by both phagocytic (J774A.1 macrophages) and non-phagocytic cells (CHO-K1) through activation of different entry pathways depending on the cell type^32^. In the context of infection by *B. pertussis* it is thought that upon ACT secretion an “atmosphere” of active toxin molecules is formed around the bacteria^33^. In the present study, we sought to determine whether the ACT molecules surrounding the bacteria might be able to induce the internalisation of *B. pertussis* into non-phagocytic cells. For this purpose, we employed two bacterial strains, *B. pertussis* strain BP18323 which expresses the *bgv*-regulated virulence factors including ACT^34^, and a non-virulent strain that lacks the *bgv* determinant, and therefore cannot express the *bgv*-regulated virulence factors (strain BP18323H^-^)^34^. We “coated” the non-virulent bacteria (BP18323 H^-^) with the toxin by attaching purified ACT to the cell surface, and to assess invasion we incubated non-phagocytic CHO-K1 cells with the “toxin-coated” bacteria. We show here that purified ACT is sufficient to promote internalisation of live bacteria into non-phagocytic CHO-K1 cells, and that internalised bacteria survive within the cells.

## Results

### ACT and *B. pertussis* induce cellular actin rearrangements

Bacterial uptake is normally preceded by perturbations of the cellular cytoskeleton, as documented for the invasive pathogenic species *Listeria monocytogenes* and *Salmonella typhimurium*^35^*. B. pertussis* can invade non-phagocytic epithelial cell lines and professional phagocytic cells (e.g. macrophages and neutrophils)^6^,^7^,^8^,^9^,^10^,^11^. We therefore explored the effect of ACT on the cell architecture. ACT toxin can bind and intoxicate, with different efficiency, a variety of cell types, including both macrophages and neutrophils which express the specific ACT receptor αMβ2 integrin^22^, as well as cells that do not express it^30^. The αMβ2 integrin is a bona fide phagocytic receptor for professional phagocytes with a central role in microbial uptake^36^,^37^,^38^; ligand binding to the integrin may activate phagocytosis^38^. Therefore, we used here CHO-K1 cells to discern whether ACT *per se* (not the toxin-integrin interaction) is able to activate signaling that promotes bacterial endocytosis. Besides, the non-phagocytic cell line has been previously used by our laboratory for ACT characterization^32^.

**Figure 1:**
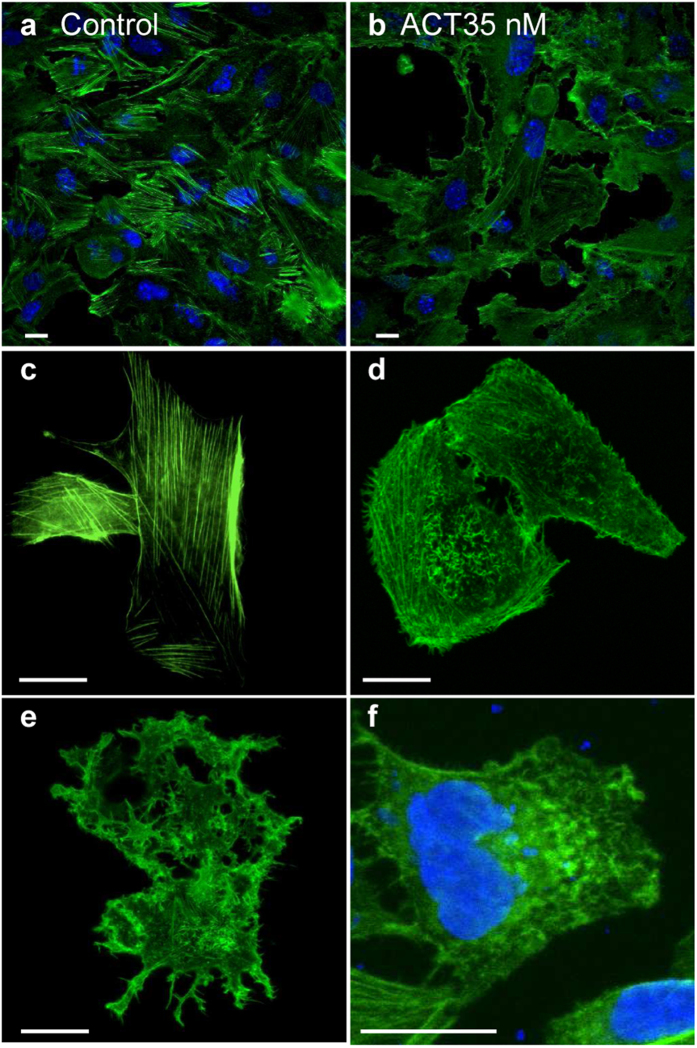
Reorganization of the cell cytoskeleton induced by ACT, or by *B. pertussis*. Exposure of CHO-K1 cells to purified ACT or to *B. pertussis* visibly affected cellular actin microfilament organization. CHO-K1 cells were treated with purified ACT or with *B. pertussis*, fixed, permeabilized and stained with Alexa Fluor® 488 phalloidin to visualize actin cytoskeleton and with Hoechst to visualize DNA as described in *Experimental procedures*. **(a)** Control. Untreated CHO-K1 cells at low magnification; **(b)** CHO-K1 cells incubated with 2 μg/mL ACT, low magnification view; **(c)** More detailed view of control, untreated CHO-K1 cells shown at higher magnification; (**d**) Detailed view of CHO-K1 cells incubated with 2 μg/mL ACT and shown at higher magnification; (**e**) Detailed view of CHO-K1 cells incubated with 5 μg/mL ACT **(f)** Detailed view of CHO-K1 cells incubated with *B. pertussis* (m.o.i 100), shown at higher magnification in order to better visualize the bacteria stained by Hoechst. Representative confocal images from three independent experiments are shown. Scale bars, 20 μm. A minimum of 30 cells were analyzed in each individual experiment.

Exposure of CHO-K1 cells to purified free ACT (2 μg/mL or 5 μg/mL, 5 min) had a prominent effect on the cellular actin cytoskeleton (Fig. 1). While control, untreated CHO-K1 cells (cells in vehicle buffer) exhibited phalloidin-stained, long actin filaments (Fig. 1c), in ACT-treated cells phalloidin-labeled actin adopted a non-fibrillar structure, and was evenly distributed in some cells (Fig. 1d). In other cells, actin localized predominantly to sub-plasma membrane positions (Fig. 1d). Membrane protrusions were also seen (Fig. 1d). This actin restructuring effect was more prominent in the cells incubated with higher ACT concentrations (Fig. 1e). A field view showing that these results are representative of a large population is shown in Fig. 1a,b.

CHO-K1 cells exposed to *B. pertussis* (multiplicity of infection (moi) of 100 bacteria per cell, similar to the used in other works^6^,^39^, adopted a morphology very similar to the cells treated with purified ACT (Fig. 1f) in which the phalloidin-bound material became evenly distributed or located underneath the plasma membrane (Fig. 1f) (Panel f of Fig. 1 is shown at a higher magnification relative to the panels a-e, to better visualise the bacteria stained by Hoechst). Cell viability remained >80% for the incubation periods tested (36 h) (not shown). These observations provided the first clue that ACT could be involved in bacterial invasion.

### “ACT-coated” non-virulent *B. pertussis* are internalised by non-phagocytic cells

To assess the possibility of bacterial internalisation following cytoskeletal rearrangement, we performed a bacterial invasion assay using the method of gentamicin survival^40^. This assay has been demonstrated to be effective in invasion assays with *B. pertussis*^6^,^39^.

As wild type *B. pertussis* produces several adhesins and toxins that could contribute to invasion, to determine whether ACT is sufficient or not to induce bacterial entry we used a *bgv*-negative mutant *B. pertussis* (strain BP18323H^-^) that cannot express *bgv*-regulated virulence factors, including ACT. These bacteria were coated with purified ACT toxin as described in *Materials and Methods.* Typically, 1 × 10^6^ bacteria were initially incubated with 20 μg/mL of ACT, and after washing the unbound protein, resultant ACT-coated bacteria had ≈0.55 ± 0.31 μg of toxin/10^6^ bacteria. The catalytic activity of bacteria-bound ACT was tested (*Materials and Methods*), and it was found that the enzymatic activity of bacterial preparations containing different amounts of bound toxin in the range 0.04–0.71 μg, was linearly proportional to the amount of toxin bound to the bacteria (Supplementary Figure S1). We designated these treated bacteria “**ACT-coated bacteria**”, and employed them in subsequent experiments.

**Figure 2:**
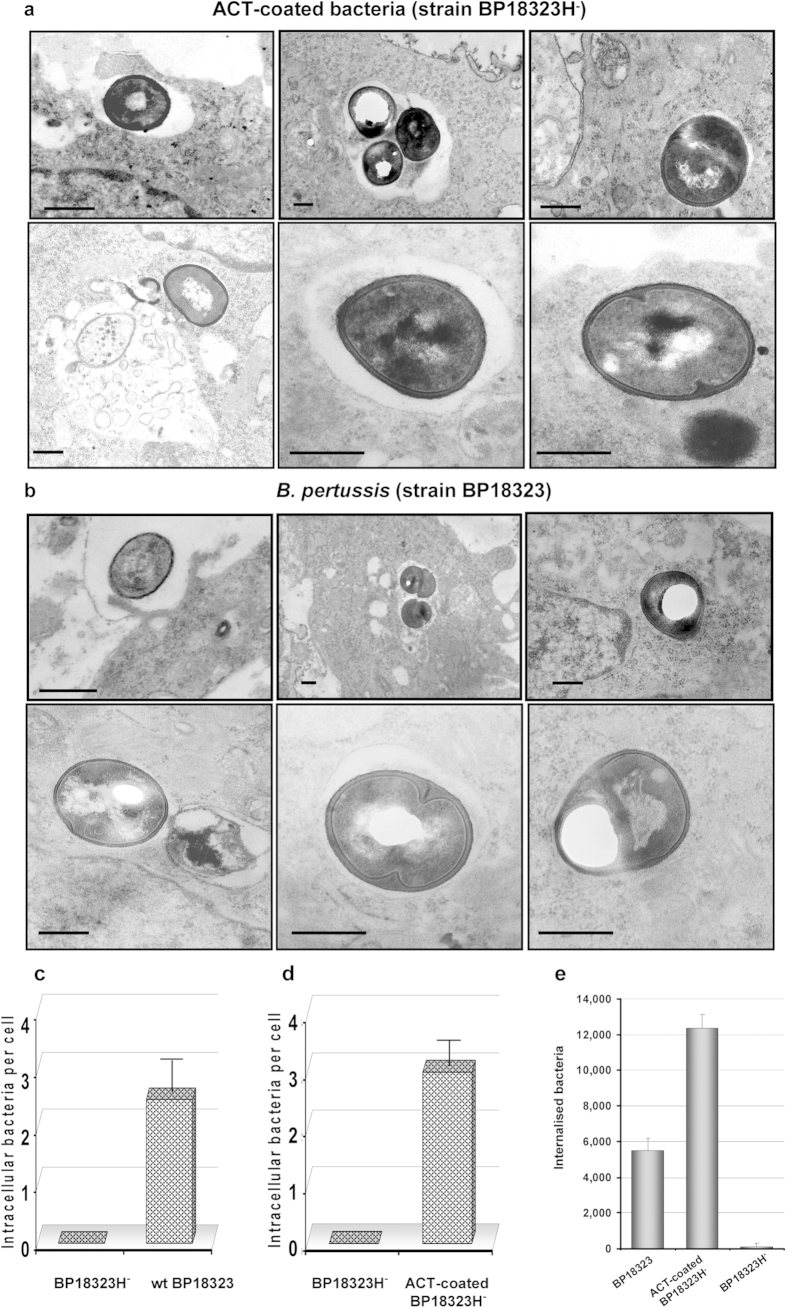
“ACT-coated bacteria” or virulent *B. pertussis* are taken up by non-phagocytic cells. Incubation of CHO-K1 cells with “ACT-coated bacteria” **(a)** or with virulent, parental *B. pertussis* **(b)**, results in bacterial internalisation, as visualized by electron microscopy. A detailed analysis of electron micrographs taken from infected CHO-K1 cells shows the different steps of the invasion process: first the bacteria were attached onto the cell membrane (upper left panel, **2a**,**b**), then internalized, with some of the bacteria appearing inside vesicles, surrounded by a membrane (upper middle and upper right panels, **2a**,**b**), and some of them appearing “free” in the cytosol (lower panels, **2a**,**b**). Representative images of three independent experiments are shown. Scale bars, 0.5 μm. The bacterial invasion assay and electron microscopy analysis were performed as described in *Experimental procedures*. Panels (**c**,**d)** show respectively, the extent of bacterial internalisation quantified from cells treated with virulent parental *B. pertussis* (wt BP18323) relative to non-virulent strain (BP18323H^-^) and expressed as number of intracellular bacteria per cell, or from cells incubated with “ACT-coated bacteria” (BP18323H^-^+ACT) relative to non-virulent non-coated strain (BP18323H^-^), and expressed as number of intracellular bacteria per cell in both cases. Panel (**e**) shows the total number of internalized bacteria quantified in the three different cases after 2h of invasion.

CHO-K1 cells were exposed to these “ACT-coated bacteria” (moi 100 bacteria per cell) and the invasion assay was performed. We visualised by electron microscopy ultrathin sections of ~100 CHO-K1 cells at various stages of bacterial internalisation (Fig. 2a). The bacteria were first adsorbed onto the cell membrane, developing contacts with the target cell and membrane extensions were visible close to the bacteria (Fig. 2a, left upper panel). In other sections, cells containing intracellular bacteria inside endosomes were observed (Fig. 2a, middle panel). At higher magnification it can be observed both intracellular bacteria surrounded by the endosome membrane and “free” bacteria that were devoid of a surrounding membrane (Fig. 2a, middle and right lower panel).

**Figure 3:**
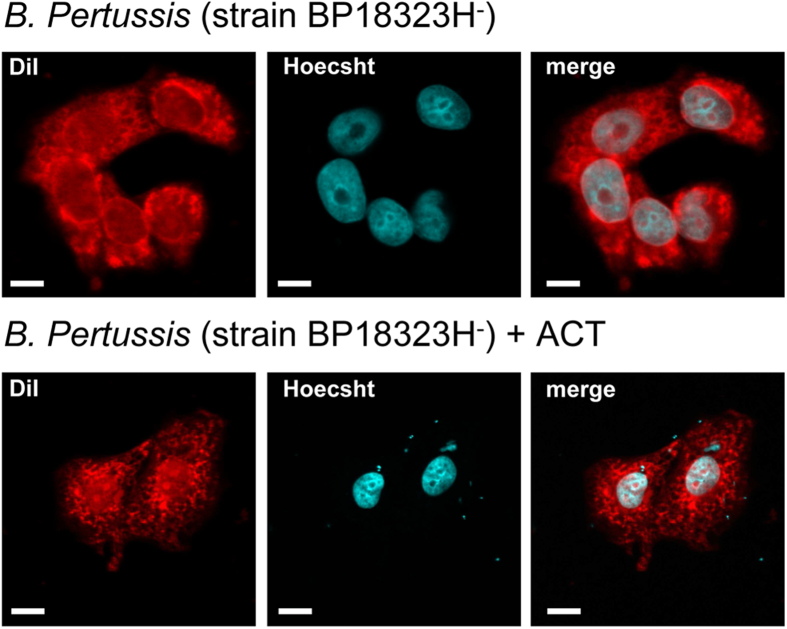
Low magnification confocal images of CHO-K1 cells incubated with non-virulent *B. pertussis* (strain BP18323H^-^) or “ACT-coated bacteria”. CHO-K1 cells were incubated with non-virulent *B. pertussis* (strain BP18323H^-^) (**a**) or with “ACT-coated bacteria” (**b**) and invasion assay and confocal microscopy analysis were performed as described in *Experimental procedures*. As observed in the images the non-virulent *B.p*. strain, which does not express nor ACT nor adhesins, was hardly found in the cell interior (**a**). In contrast, the non-virulent bacteria that are coated with ACT can be found in the interior of CHO-K1 cells. Cell membranes were stained with the fluorescent probe DiI (*red*) and DNA with Hoechst (*blue*). Representative images of three independent experiments are shown. A minimum of 30 cells were analyzed in each individual experiment. Scale bars, 20 μm.

To verify that parental *B. pertussis* (BP18323 strain) naturally expressing ACT was also capable to enter these cells and in order to have a reference of the efficiency of the internalisation relative to the “ACT-coated bacteria” we performed the invasion assay with the virulent *B. pertussis* (BP18323 strain) (moi 100 bacteria per cell). The analysis by electron microscopy of ultrathin sections of infected CHO-K1 cells are shown in Fig. 2b. Similar steps of internalisation were observed for the wild type bacteria, with bacteria first adsorbed onto the cell membrane (Fig. 2b left upper panel), and internalised bacteria inside endosomes (Fig. 2b middle upper panel) as well as “free” bacteria in the cytosol (Fig. 2b right panels), similarly to the observations with “ACT-coated bacteria” (Fig. 2a). Low magnification confocal microscopy images of CHO-K1 cells incubated with “ACT-coated bacteria” show several cells containing one or more “ACT-coated bacteria” inside (Fig. 3), in contrast to the cells that were incubated with control “non-coated” bacteria (non-virulent BP18323H^-^ strain, without ACT bound) which showed negligible bacterial internalisation (Fig. 3). To compare the efficacy of internalisation between the virulent parental *B. pertussis* (wt BP18323) and the “ACT-coated bacteria” (BP18323H^-^+ACT) we quantified the number of intracellular bacteria for both cases. Data are expressed as number of intracellular bacteria per cell (Fig. 2c,d). For the parental *B. pertussis* strain ≈2 bacteria per cell were quantified (Fig. 2c), while for the “ACT-coated bacteria an average of ≈3 bacteria per cell were determined (Fig. 2d). For the control non-coated non-virulent bacteria, almost no bacteria were found inside the cells. These values were rather similar to the values found by Lamberti *et al.* in macrophages (≈2 bacteria per cell)^11^, or in epithelial cells (≈5 bacteria per cell)^41^.

**Figure 4:**
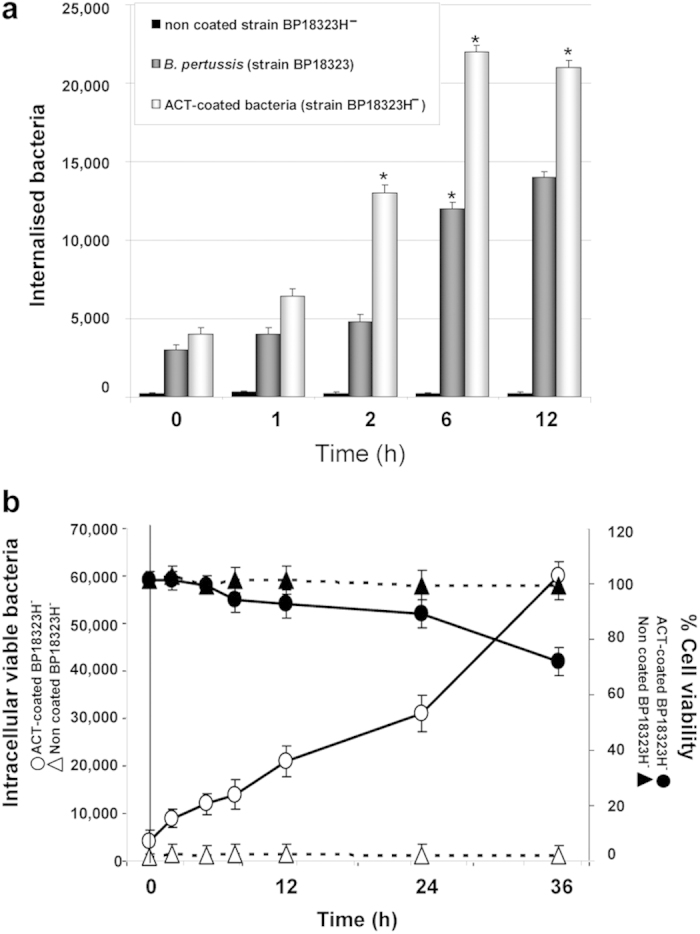
Quantification of the total number of internalized bacteria and viability of the bacteria-containing cells. CHO-K1 cells were incubated with control non-virulent *B. pertussis* (BP18323H^-^), with virulent *B. pertussis* (strain BP18323) or with “ACT-coated bacteria”, and invasion was followed as described in *Experimental procedures*. The number of internalized bacteria at different times of invasion (0.5–12 h) was quantified as described in *Experimental procedures.* Time =0 h represents the invasion occurring after 2 h cell incubation with bacteria, washing and killing of extracellular bacteria by gentamicin **(a)**. Cell viability of CHO-K1 cells and number of internalized bacteria were determined for each time-point (0.5–36 h) as described in *Experimental procedures* **(b)**. Internalisation of “ACT-coated bacteria” only induced a slight to moderate decrease in the cell viability of the infected CHO-K1 cells, and most of the internalized bacteria could grow after infection. Data shown are the mean ± SD of at least three independent experiments performed in triplicate, with *p < 0.01.

Quantification of the total number of intracellular bacteria at different times after invasion revealed that there were fewer virulent bacteria (strain BP18323) than “ACT-coated bacteria” inside cells (Fig. 4a), suggesting that other toxic effectors expressed by the parental bacterium reduced invasiveness, or, perhaps more likely, induced a higher rate of cell mortality. To test this hypothesis we performed a cytotoxicity assay (LDH release) and quantification of cAMP in the cells incubated with one or other bacterial strain. The cAMP concentration in cells incubated with parental *B. pertussis* was significantly higher than in cells incubated with “ACT-coated bacteria “(Supplementary Fig. S3) indicating that the higher intoxication caused by the parental bacteria may kill CHO-K1 cells. Higher cell mortality was indeed confirmed for the cells that had been incubated with the parental *B. pertussis* (Supplementary Figure S4). The efficacy of bacterial uptake after 6 h incubation was 1.2 ± 0.1% for parental *B. pertussis* and 2.2 ± 0.1% for the “ACT-coated bacteria”. Similar values (0.4–2.8%) had been reported by others in invasion assays with *B. pertussis* in macrophages^11^ and in epithelial cells^41^. Importantly, invaded CHO-K1 cells remained viable for at least 36 h although an increasing number of live bacteria were observed inside cells suggesting that the internalised bacteria can survive and multiply in the invaded cells (Fig. 4b). The set of data shown in Figs 2, 3, 4 suggested that ACT can promote *B. pertussis* internalisation.

### Characterization of the entry route of ACT-coated *B. pertussis*

**Figure 5:**
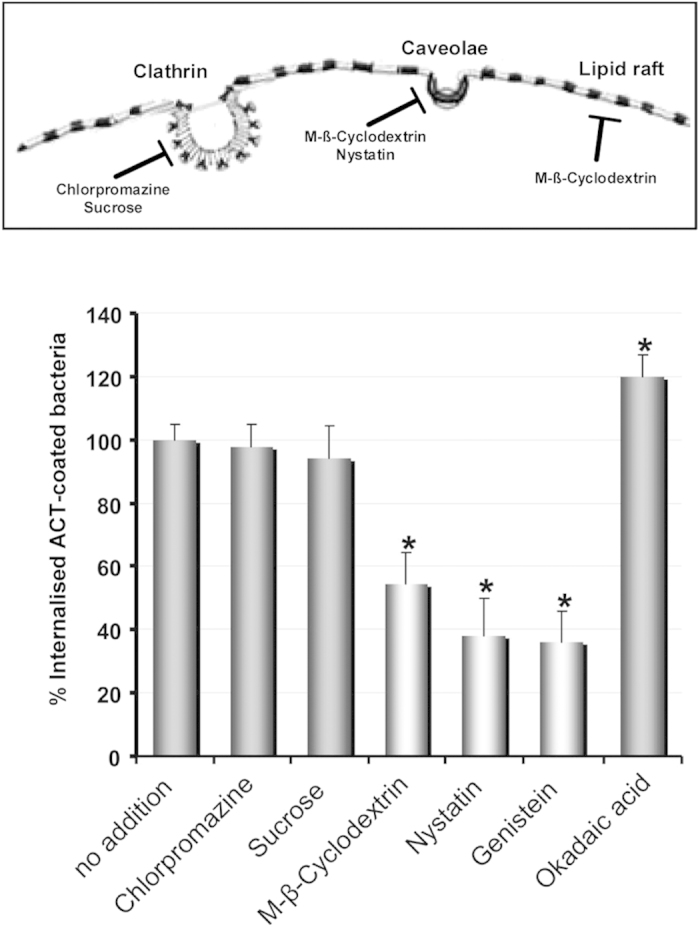
Characterization of the invasion pathway of “ACT-coated bacteria”. Several inhibitors of the different possible entry routes were analyzed and it was concluded that “ACT-coated *B. pertussis”* exploits a clathrin-independent, cholesterol-dependent (caveolae-dependent) entry pathway to invade CHO-K1 cells, and that it requires the activation of tyrosine kinases. CHO-K1 cells were pre-incubated with chemical inhibitors (5 μg/mL chlorpromazine, 450 mM sucrose, 10 mM methyl-β-cyclodextrin, 0.25 μg/mL nystatin, 100 μM genistein and 1 μM okadaic acid) for 30 min at 37 °C, then cell invasion was assayed as described in *Experimental procedures.* Chlorpromazine, sucrose and methyl-β-cyclodextrin were dissolved in DMEM, nystatin, genistein and okadaic acid, were dissolved in DMSO. Controls showing that vehicle has no effect on internalisation are shown in Fig. S5. The data were normalized to the control sample (*no addition*) and expressed as per cent of control entry. Data shown are the mean ± SD of at least three independent experiments performed in quintuplicate, with *p < 0.01.

To determine the main features of the entry pathway followed by the “ACT-coated bacteria” we pre-treated CHO-K1 cells with several endocytosis inhibitors and performed the invasion assay. The common inhibitors of clathrin-mediated endocytosis (5 μg/mL chlorpromazine or high-sucrose medium) did not significantly affect invasion (Fig. 5), while treatment with 10 mM methyl-β-cyclodextrin, a drug that disrupts cholesterol-enriched membrane domains, reduced bacterial uptake by ~50% (Fig. 5). Nystatin (0.5 μg/mL), another cholesterol-depleting compound, exerted an even more profound inhibitory effect on bacterial uptake (Fig. 5), suggesting that cholesterol-rich domains may be involved in the invasion process, a hypothesis that is in full agreement with previous data obtained with purified ACT, in which it was found that cholesterol is involved in the endocytosis of the toxin itself by macrophages and CHO cells^32^,^42^. Vehicle controls using DMEM or DMSO did not cause any difference in internalisation (Supplementary Figure S5).

In the lipid-raft dependent invasion processes of other pathogens, activation of tyrosine kinases has been found to be crucial^43^,^44^. For ACT we had previously observed that its internalisation depends on the activation of this family of kinases^42^, so we next studied their contribution to invasion by *B. pertussis*. Pre-incubation of the CHO-K1 cells with the tyrosine-kinase inhibitor genistein (100 μM) inhibited invasion about 60% (Fig. 5). Okadaic acid (1 μM), which inhibits tyrosine phosphatases, had the opposite effect of genistein (Fig. 5), indicating that activation of tyrosine kinases is also critical for invasion by *B. pertussis*. Thus, the “ACT-coated bacteria” are endocytosed by a clathrin-independent, raft-dependent and tyrosine kinase dependent pathway.

### Characterization of bacteria-containing endosomes

**Figure 6:**
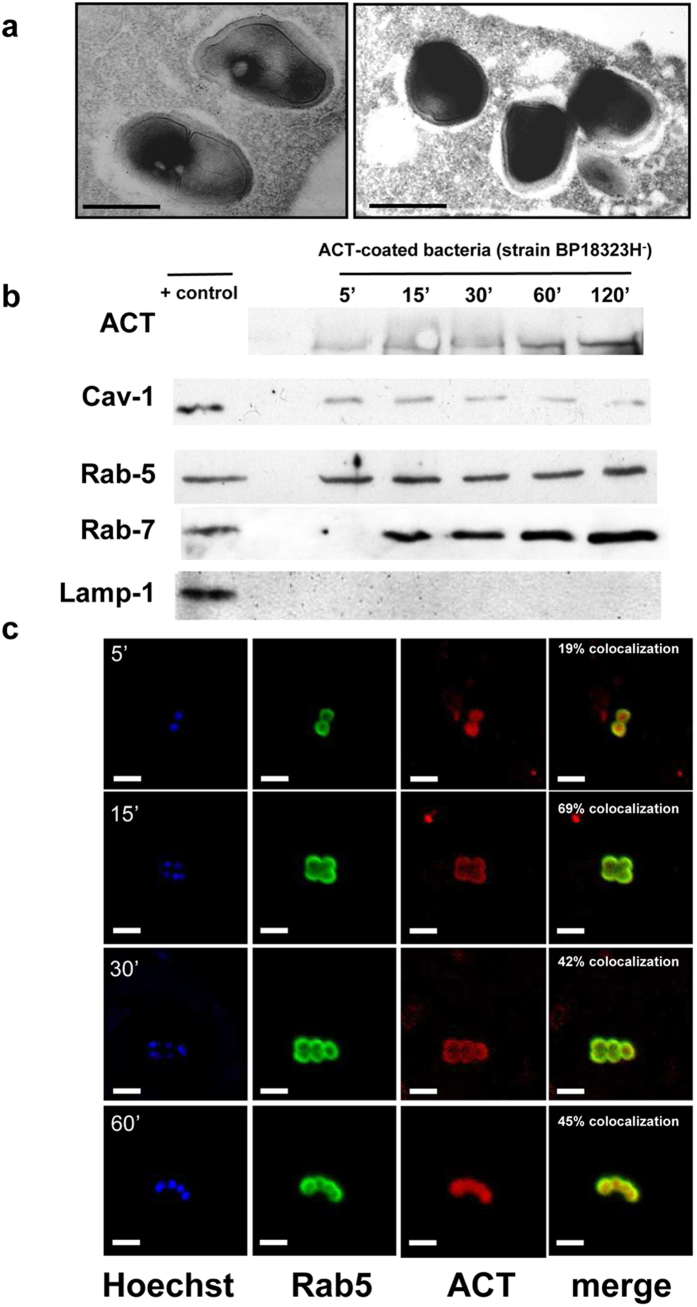
Characterization of bacteria-containing endosomes. Ultrathin cuts of infected cells (**a**) or endosome membranes isolated from the infected cells at different times of incubation (5–120 min) and submitted to SDS-PAGE electrophoresis **(b)**, or purified endosomes isolated and purified from the infected cells **(c)**, were respectively analysed by electron microscopy **(a**), by Western blot **(b)** or by confocal microscopy **(c).** Common markers of the different endocytic stages were used for the respective analysis: gold-labelled anti-**Rab-5** Ab for the electron microscopy **(a)**, or anti**-Rab-5** Ab, as early endosome marker; anti- **Rab7** Ab as late endosomal marker, anti-**LAMP-1** Ab as lysosomal marker, and anti-**Cav-1** Ab as caveolae marker, in the Western blot assay **(b)**. For confocal analysis DNA was stained with Hoechst, Rab-5 was stained with a FITC-labelled secondary Ab, and ACT was stained with a Texas Red®-labelled secondary Ab. Quantification of signal co-localization is given **(c)**. From the combination of all the data it was concluded that “ACT-coated *B. pertussis”* resides in Cav-1 positive, Rab-5 and Rab-7 positive and LAMP-1 negative non-acidic compartments in the infected CHO-K1 cells. Representative images of three independent experiments are shown. Black scale bars, 1 μm; white scale bars, 2.5 μm.

Detection of intracellular viable bacteria upon CHO-K1 cell invasion (Fig. 3b) suggested that classical endolysosomal fusion is likely avoided in the entry pathway followed by ACT-coated-bacteria. To explore this possibility we evaluated the features of bacteria-containing endosomes; endosome-containing intact CHO-K1 cells as well as purified endosomes isolated from CHO-K1 cells infected with “ACT-coated bacteria” were studied by different techniques (Fig. 6). Electron microscopy of ultrathin sections of invaded CHO-K1 cells (Fig. 6a) revealed that *B. pertussis*-containing vesicles were Rab-5 positive (early endosomal marker) for as long as 2 h, as determined by the use of anti-Rab-5 immunogold-labeled antibodies (Fig. 6a). The same samples were LAMP-1 negative (lysosomal marker) at all tested time points (Fig. 6a), supporting the hypothesis that the vesicles containing “ACT-coated bacteria” were non-acidic.

Immunoblotting of the membrane fractions of endosomes purified from the infected CHO-K1 cells after different periods of infection (5–120 min) revealed the simultaneous presence of ACT, Cav-1 (a major protein forming membrane caveolae), and Rab-5 (The same amount of protein was loaded in each case) (Fig. 6b). The purified endosomes also contained Rab7, a marker of late endosomes, but were negative for the lysosomal marker Lamp-1, thus corroborating the electron microscopy data. Confocal microscopy of endosomes purified from the infected cells (Fig. 6c) also corroborated the co-localisation of ACT and Rab-5 on the membranes of bacteria-containing endosomes. Bacterial DNA was stained with the fluorescent marker Hoechst in order to visualise bacteria in the endosomes (Fig. 6c). Results very similar to those shown in Fig. 6c were obtained by infecting the CHO-K1 cells with the parental virulent *B. pertussis* (Supplementary Fig. S6).

Analysis of the lipid composition of purified endosomal membranes confirmed a high content of cholesterol and sphingomyelin in these membranes (Table 1), in good agreement with our previous observation that methyl-β-cyclodextrin and nystatin, two cholesterol-depleting agents, substantially inhibit bacterial invasion (Fig. 6).

### ACT is sufficient to promote cell invasion

**Figure 7:**
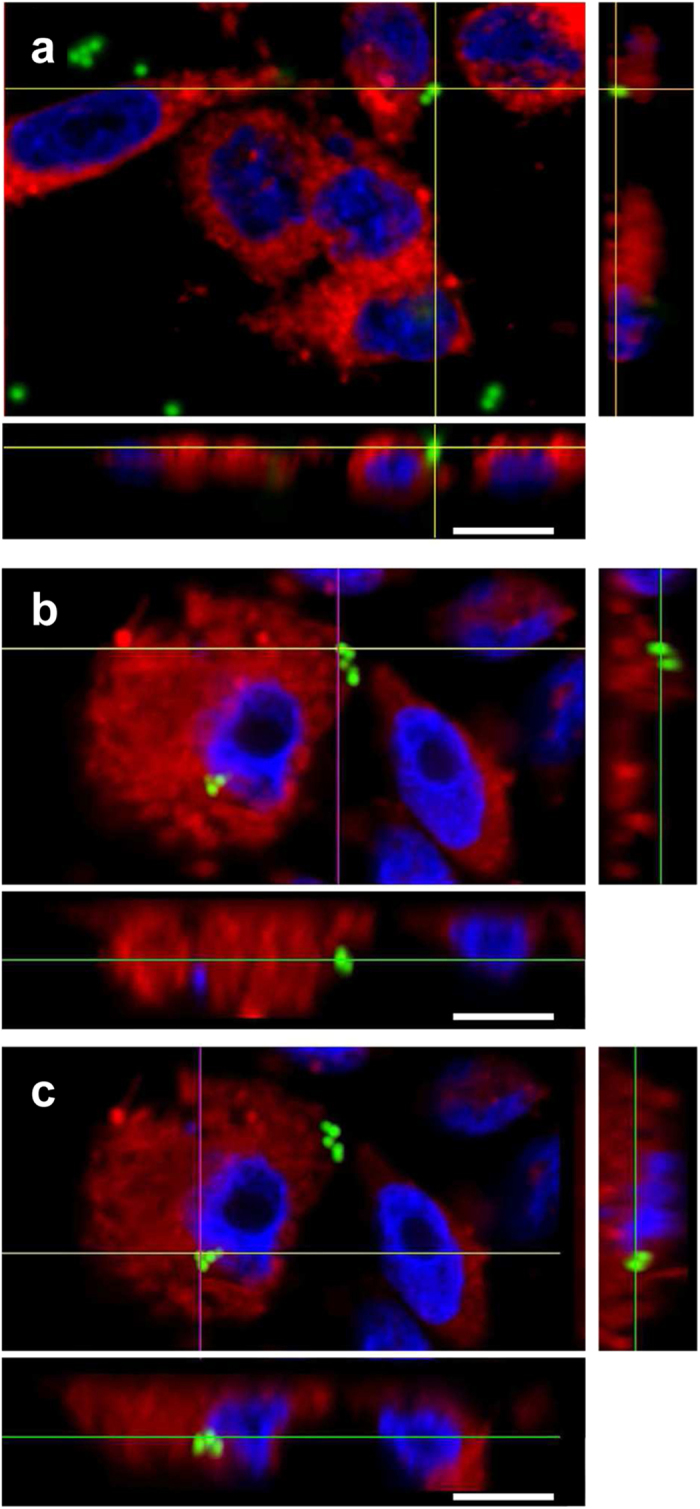
ACT is sufficient to promote internalisation of “toxin-coated beads”. CHO-K1 cells were incubated with fluorescent “ACT-coated beads” (green fluorescence) and invasion assay and confocal microscopy analysis were performed as described in *Experimental procedures*. In control cells treated with BSA-coated beads the fluorescent beads were outside the cells **(a)** Analysis of the microscopy images revealed the presence of some fluorescent “ACT-coated” beads just “attached” or adhered to cell membranes **(b)** while other beads were clearly detected inside the cells **(c)**. For Z-stack confocal analysis, cell membranes were stained with DiI (*red*) and DNA with Hoechst (*blue*). Representative images of three independent experiments are shown. Scale bars, 10 μm. A minimum of 30 cells were analyzed in each individual experiment.

Fluorescent polymeric particles (latex beads) coated with ACT were used to unambiguously determine whether ACT, in the absence of any other bacterial factor, is sufficient to induce bacterial invasion of non-phagocytic cells. To test this hypothesis our invasion assay was modified to include green fluorescent beads of 1 μm in diameter (similar to the size of *B. pertussis*) externally coated with purified ACT (following a protocol similar to that used to coat live bacteria). Identical particles coated with bovine serum albumin (BSA) instead of ACT were used as a negative control of invasion. Confocal z-stack images of CHO-K1 cells incubated with ACT-coated or BSA-coated beads were taken (Fig. 7). In this assay with fluorescent beads, to ensure that the ACT-coated beads are inside the cells, internalisation was visualised at high resolution and by z-stack image acquisition, due to the impossibility to quench the fluorescence signal of the fluorescent beads attached to the cell membrane. Cell nuclei were stained with Hoechst. ACT-coated beads adhered to the cell surface (Fig. 7b), likely before their internalisation, and the green fluorescence signal corresponding to engulfed ACT-coated beads was clearly visible in the interior of the cells (Fig. 7c). In the negative control, this is, cells incubated with BSA-coated beads (Fig. 7a ), the green fluorescence was only visible outside the cells, in some cases attached to the cell surface (Fig. 7a), suggesting that ACT was necessary and sufficient to promote the engulfment of the latex beads by CHO-K1 cells. We conclude that ACT by itself promotes the bacterial invasion of non-phagocytic cells.

### Ca^2+^ influx is required for the bacterial uptake

**Figure 8 f8:**
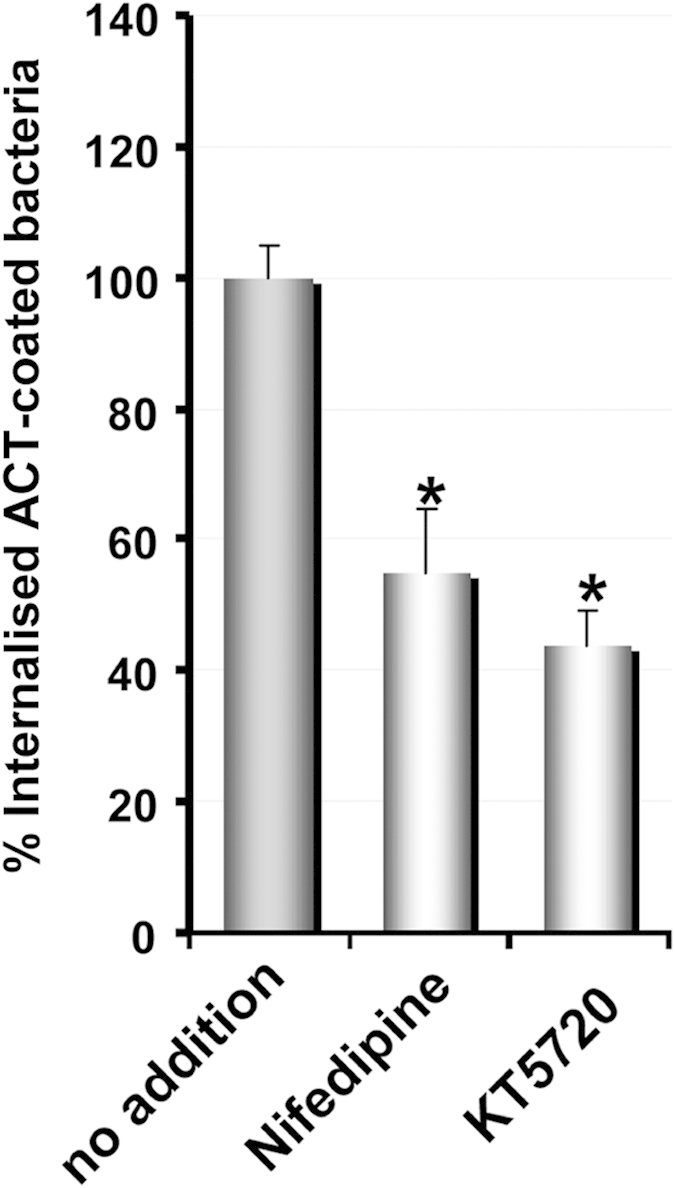
ACT-induced Ca^2+^ influx is necessary for the uptake of the “ACT-coated bacteria”. CHO-K1 cells were pre-incubated for 30 min at 37 °C with nifedipine (10 μM), an inhibitor of L-type Ca^2+^ channels, or with KT5720 (56 nM), which specifically inhibits cAMP-dependent protein kinase A (involved in the activation of L-type channels), then the pretreated cells were exposed to “ACT-coated bacteria”, and the invasion assay was performed as described in *Experimental procedures.* The data were normalized to the control cells (no inhibitor addition) and expressed as per cent of control entry. Data shown are the mean ± SD of at least three independent experiments performed in quintuplicate, with *p < 0.01.

In previous work from this laboratory we had observed that the internalisation of purified ACT required elevation of intracellular Ca^2+^ 45. We had also previously noted that this toxin induces rapid Ca^2+^ influx through activation of PKA-dependent L-type Ca^2+^-channels^45^. Hence, we explored whether Ca^2+^ was also necessary for the ACT-mediated bacterial uptake, and for that we used two pharmacological inhibitors. Pre-incubation of the CHO-K1 cells with nifedipine (10 μM), inhibitor of L-type Ca^2+^ channels, reduced bacterial entry by ~50% (Fig. 8). Pre-treatment of CHO-K1 cells with KT5720 (56 nM), which specifically inhibits cAMP-dependent protein kinase A (involved in the activation of L-type channels), resulted in a similarly decreased invasion (Fig. 8). These results strongly suggest that Ca^2+^ influx induced by ACT is necessary for the uptake of the “ACT-coated bacteria”.

## Discussion

[Fig f8]A number of studies have provided evidence that *B. pertussis* is capable of cell invasion using both primary cell cultures and cell lines^6^,^7^,^8^,^9^,^10^,^11^. However the precise mechanism used by *B. pertussis* for cell entry, or the putative bacterial factors involved in invasion are not yet fully understood. In this work we have found that adenylate cyclase toxin (ACT), a major virulence factor secreted by *B. pertussis*, promotes invasion of this pathogenic bacterium into CHO-K1 non-phagocytic cells, in a Ca^2+^- and Tyr-kinase-dependent manner (Fig. 8).

ACT, as other members of the RTX family, is a large hydrophobic-amphipathic toxin, with a high tendency to aggregation. In the context of *B. pertussis* infection it has been suggested that ACT proteins might form an atmosphere of active toxin molecules around the bacterial cell^46^. Here we have somehow “mimicked” this situation by “coating” the surface of live bacteria with low ACT concentrations. The *B. pertussis* strain used here for ACT-coating (strain BP18323H^-^) does not express *bgv*-regulated virulence factors, including filamentous hemagglutinin (FHA), which was reported to retain ACT on the bacterial surface by physical association with it hindering ACT from reaching and intoxicating the target cell^47^,^48^. In the “ACT-coated” bacteria used here the toxin attached to the bacterial surface is cAMP-producing and thus, active. We propose that ACT is associated to the bacterial surface through hydrophobic and/or electrostatic interactions mediated by its hydrophobic and RTX domains, and that this weak association keeps or preserves ACT in an active conformation, allowing its detachment from the bacterial outer membrane and subsequent insertion into close host cell membranes, eventually leading to ACT-mediated bacterial invasion.

For known invasive bacterial pathogens, bacterial entry into non-phagocytic cells relies on the binding of bacterial factor(s) to component(s) of the host cell surface. For *Shigella flexneri*, the interaction of Ipa proteins with α5β1 integrin promotes entry of the bacteria into epithelial cells^49^. In the case of *Yersinia*, the outer membrane protein invasin binds to β1 integrin receptors, leading to bacterial entry^50^. For *Listeria monocytogenes* host E-cadherin serves as a receptor for the bacterial protein internalin (InlA) to enter human enterocyte-like epithelial cell line Caco-2 and some other epithelial cells^51^. In sharp contrast, we find here that ACT-mediated bacterial internalisation does not necessarily require a toxin-receptor interaction, as both the virulent parental *B. pertussis* BP18323 bacteria and the non-virulent ACT-coated bacteria (BP18323H^-^ strain), and even ACT-coated latex beads are similarly taken up by CHO-K1 cells, a non-phagocytic cell line that does not express the ACT receptor, the β2 integrin CD11b/CD18. Consistent with this, it was observed that ACT itself is internalized both by receptor-bearing cells^32^ and by cells that do not express the CD11b/CD18 toxin-receptor^32^, suggesting that the hydrophobic-amphipathic nature of ACT is enough to allow productive attachment to a variety of host cell membranes.

Consistent with the idea that bacterial uptake requires host cytoskeleton rearrangements including F-actin or microtubules^52^ we observe here that the cell actin cytoskeleton is prominently remodeled upon contact with parental *B. pertussis*, with a visible destruction of actin filaments and the formation of membrane protrusions. A very similar effect, that is concentration dependent, is also observed upon incubation of CHO-K1 cells with purified ACT, thus suggesting that the toxin itself triggers the required signals to rearrange the actin filaments. Restructuring of the cell actin cytoskeleton may be caused by different signaling events. Modifications in the intracellular Ca^2+^ and cAMP-mediated signalling have been involved in perturbations of the actin cytoskeleton homeostasis in different cells^53^,^54^. ACT generates rises in cAMP levels in the cytosol of the target cells^17^ and induces intracellular Ca^2+^ rises^45^, thus both factors may be involved in the effects on the cytoskeleton observed here. In support of this we observe an important reduction in bacterial entry under conditions in which the ACT-induced Ca^2+^-influx is inhibited.

We find that ACT-coated bacteria follow a cholesterol-dependent, caveolae-dependent entry pathway in which bacteria-containing vesicles show markers of early and late endosomes (Cav-1-positive, Rab-5- and Rab-7-positive) but appear to avoid ulterior fusion with lysosomes (LAMP-1-negative for all tested times). This mechanism is part of the virulence strategies of several invasive bacteria, allowing them to evade intracellular death^43^. Our observations are in agreement with earlier studies indicating that *B. pertussis* survives in non-acidic compartments of human macrophages^11^ or in respiratory epithelial cells^41^.

We show evidences that internalisation of ACT-coated bacteria shares several features with the endocytosis of the purified toxin, namely the requirement for Ca^2+^-influx and the involvement of tyrosine kinases^32^. This suggests that a similar common entry route is activated and is involved in both processes. Tyrosine kinases are key signaling molecules involved in diverse processes of nucleated cells. In the internalisation of the purified ACT by the CHO-K1 cells we discovered that phosphorylation of key components of the endocytic machinery involved in the caveolae-mediated ACT uptake is required^32^. Recently, tyrosine kinases have been involved in F-actin restructuring at the *Listeria monocytogenes* entry site^55^, suggesting that these signaling proteins may be key collaborators in bacterial internalisation processes. Ca^2+^ influx was also reported to be essential for *L. monocytogenes* entry into nonphagocytic cells^55^.

Determination of the intracellular bacteria taken up by CHO-K1 cells (2–3 bacteria per cell) indicates that ACT-mediated internalisation of *B. pertussis* is not as efficient as the entry of other well known “invasive” bacteria such as *Listeria* or *Salmonella.* Nevertheless, pathogenic *B. pertussis* produces other factors such adhesins which have been reported to contribute to invasion^10^ and that might increase the efficiency of bacterial uptake *in vivo*. Human cell invasion may not be the main *B. pertussis* pathogenic pathway, but the fact that it expresses virulence factors such as ACT that are able to induce the engulfment of bacteria led us to speculate that during the infection process non-phagocytic cells could be a favorable medium to evade the host immune system, increasing the viability and persistence in the host.

In contrast to invasion by the invasive bacterium *Listeria monocytogenes* and other bacterial species^56^, the clathrin-mediated endocytosis machinery does not seem to be involved in the ACT-promoted engulfment of *B. pertussis*, at least in the non-phagocytic cells (CHO-K1 cells) tested here. However we cannot discard the possibility that in immune cells such as neutrophils and macrophages (which express the CD11b/CD18 integrin), a clathrin-dependent route will be involved in the uptake of ACT-coated bacteria, as we previously observed for the endocytosis of purified ACT by J774A.1 macrophages^42^.

ACT is a hemolysin, thus the presence of “nude” free bacteria devoid of a vesicular membrane in the target cell interior led us to hypothesize that the endosome membranes might be directly permeabilized by ACT, allowing the entrapped bacteria to escape from the internalisation vesicles. In agreement with this hypothesis we found that the endosomes purified from the infected cells were permeable to the dye DAPI which penetrates and stains the bacterial DNA, strongly suggesting that the endosomal membranes are permeabilized by ACT. Lipid analysis of the bacteria-containing vesicle membranes purified from the infected cells reveals a high content of sphingomyelin and cholesterol, two lipids typically found in membrane raft-domains. In previous investigations we determined that cholesterol facilitates ACT-induced permeabilization of liposomes^57^. This suggests that cholesterol-rich domains may favor the ACT permeabilizing activity in the endosome membrane.

Few pathogenic bacterial species have developed the capacity to induce their own uptake into non-phagocytic cells, which confers significant advantages for pathogenesis, survival, or both. The observation that *B. pertussis* employs such a mechanism of cell entry suggests that it offers a selective advantage, for example extended protection from the host’s immune system to allow the protracted release of toxins or effectors and to maintain a persistent infection. Given that ACT is highly immunogenic^58^, we think that the inclusion of ACT into new vaccine preparations and the design of specific inhibitors to block ACT-promoted invasion are promising steps for future whooping cough treatments.

## Methods

### Antibodies and reagents

Anti-adenylate cyclase toxin RTX domain mouse monoclonal antibody (MAb 9D4) was from Santa Cruz Biotechnology (Santa Cruz, CA, USA); anti-Rab-5 and anti-LAMP-1 from Cell Signalling technology (USA); anti-caveolin-1, sucrose, ficoll, methyl-β-cyclodextrin, nystatin, nifedipine, chlorpromazine, and genistein from Sigma-Aldrich (St Louis, MI, USA); KT5720, calpeptin, and Okadaic acid from Calbiochem (Merck, Germany); Fluospheres^®^ sulphate microspheres (latex beads), Hoechst, anti-mouse Texas Red^®^, anti-rabbit FITC, Vibrant DiI and Alexa Fluor^®^ phalloidin from Invitrogen, Molecular Probes (Carlsbad, CA, USA). Bacto^TM^ proteose peptone, Difco^TM^ Bordet Gengou Agar base from BD Biosciences (Spain). Gentamicin was from Gibco Thermo Fisher Scientifics, USA.

### Bacterial strains and culture conditions

Bacterial strains used in this study (*B. pertussis* 18323 H and 18323 H^-^) were cultured on BG agar (BD Biosciences, Spain) supplemented with 15% defibrinated sheep blood (Microlab-Pronadisa, Laboratorios Conda, Bilbao, Spain) for 48 h at 37 °C. The two bacterial strains were kindly provided by Dr. Nicole Guiso, Institute Pasteur, Paris, France.

### Cell culture

CHO-K1 cells (ATTC, number CCL-61) were cultured at 37 °C in DMEM supplemented with 10% (v/v) FBS, and 4 mM L-glutamine in a humidified atmosphere with 5% CO_2_.

### ACT expression and purification

ACT was expressed in *Escherichia coli* XL-1 blue cells (Stratagene) transformed with pT7CACT1 plasmid, kindly provided by Dr. Peter Sebo (Institute of Microbiology of the ASCR, v.v.i., Prague, Czech Republic) and purified and characterized as previously described^57^,^59^.

### Binding of purified ACT to *Bordetella pertussis* 18323 H^-^

*Bordetella pertussis* 18323 H^-^ was grown in BG liquid medium for 48 h at 37 °C under constant stirring until O.D. (650 nm) =1.0 which is equivalent to ≈2.5 × 10^7^ bact/mL, then, centrifuged at 6,000 × g for 5 min, resuspended in DMEM culture medium and incubated with different initial concentrations (5–100 μg/10^6^ bacteria) of ACT. The mixture (ACT and bacteria) was incubated in eppendorf tubes on a rotating wheel at room temperature for 1 h, then bacteria were centrifuged at 1,000 × g for 5 min and washed 5 times with fresh medium to remove unbound toxin before co-incubation with CHO-K1 cells. Quantification of the real amount of ACT bound to the bacteria (triplicate) was performed by western blot using as standard known purified ACT concentrations. For bacterial invasion assays the preparation typically contained 0.55 μg ACT bound /10^6^ bacteria (which corresponded to ≈20 μg of ACT incubated with 10^6^ bacteria). This ACT concentration was used as CHO-K1 cell viability was minimally affected during co-incubation under these conditions.

### Measurement of cAMP

cAMP produced by ACT-coated bacteria (*Bordetella pertussis* 18323 H^-^) (incubation of 5–100 μg ACT/10^6^ bacteria) was assayed for 10 min at 37 °C with 2 nM CaM in AC reaction buffer (30 mM Tris-HCl, pH 7.4, 20 mM MgCl_2_ and 100 μM CaCl_2_), then the reaction was started by addition of 5 mM ATP. After 10 min at 37 °C with continuous stirring the reaction was stopped with 0.1 M HCl. The cAMP production was calculated by the direct cAMP EIA kit (Enzo lifesciences).

### Bacterial invasion assays

Bacterial invasion assays were performed according to the method of gentamicin survival. CHO-K1 cells in DMEM were incubated with virulent *B. pertussis* strain 18323 H or ACT-coated *B. pertussis* 18323 H^-^ at a multiplicity of infection (moi) of 100 bacteria per cell and centrifuged at 700 × g for 10 minutes at 21 °C. Cells were then placed for 2 h at 37 °C and 5% CO_2_. After incubation cells were washed with DMEM, and subsequently incubated with fresh gentamicin-containing DMEM (100 μg/ml gentamicin), for an additional 1 h. Cells were repeatedly washed and then lysed with 0.1% Triton X-100. The number of viable bacteria inside the cells was assessed by plating on BG agar plates supplemented with 15% sheep blood and counting the colonies grown. The % of internalised bacteria was calculated as the number of CFU/total added bacteria x 100. Each experiment was done in triplicate, and triplicates were performed 3 or more times independently. Control experiments to assess the efficacy of antibiotic bactericidal activity were performed in parallel, briefly, samples 2 × 10^8^ bacteria were incubated with DMEM containing gentamicin (100 μg/ml) for 1 h at 37 °C and then plated on BG agar. This antibiotic exposure resulted in 99.999% decrease in CFU.

### Confocal microscopy

CHO-K1 cells were grown to sub-confluency on Permanox Lab-Tek chambers in DMEM, supplemented with 10% (v/v) FBS, L-glutamine, penicillin and streptomycin. Purified ACT (2 and 5 μ/ml) or ACT-coated bacteria (20 μ/ml) were added to the medium and incubated with the cells for 2 h. Cells were washed in phosphate buffered saline (PBS) pH 7.4, fixed in 3.7% formaldehyde, and permeabilized in the presence of acetone for 3 min at −20 °C. To visualise actin cytoskeleton and DNA cells were stained with Alexa Fluor®488 phalloidin and Hoechst, respectively. For ACT-coated latex beads cellular localisation, beads were incubated with CHO-K1 cells for 2 h, then cells were washed in PBS, fixed and cellular membranes were labelled with vibrant DiI to allow a better detection of internalised latex beads. Z-stack images were obtained in a 0.2 μm slides and fluorescent latex beads and Vibrant DiI were visualised under a 488 or 546 excitation laser beam, respectively. To visualise endosomes containing wt *Bordetella pertussis* or ACT-coated bacteria, isolated endosomes were adsorbed to polylysine-coated cover slips and then fixed in 3.7% formaldehyde, and permeabilized in the presence of acetone for 3 min at –20 °C. Endosomes were stained with anti-RTX and anti-Rab5 primary monoclonal antibodies followed by incubation with FITC-and Texas Red^®^ conjugated secondary antibodies, respectively. Cells were then incubated for 10 min with Hoechst to visualise nuclei. Samples were visualised under a confocal microscope (Olympus FV500) with sequential excitation and capture image acquisition.

### Electron microscopy and immunogold labelling

ACT-coated bacteria were added to CHO-K1 cell cultures and co-incubated for 2 h. Cells washed with PBS buffer pH 7.4 were fixed in 2% glutaraldehyde and 0.1% tannic acid in 0.1 M sodium cacodylate. After post-fixation in 1% OsO_4_ samples were dehydrated and embedded in Polarbed 814 epon resin. For immunogold labelling cells fixed in 3% formaldehyde plus 0.1% glutaraldehyde in 0.1 M cacodylate were dehydrated in ethanol, then incubated in a mixture containing equal parts (v/v) ethanol and white LR at 20 °C for 1 min, followed by 24 h in pure resin at 20 °C. After two further incubations in fresh resin, the samples were transferred into resin-filled gelatin and allowed to harden at 50 °C. Sections of 60 to 80 nm were transferred onto nickel grids coated with Formvar film, blocked in PBS with 5% BSA, and incubated with primary antibodies. After washes with PBS-BSA, the grids were placed on droplets of gold-labeled secondary antibodies and then washed. Staining for contrast was performed in a saturated aqueous solution of uranyl acetate for 5 min, followed by lead citrate for 5 s. Specimens were examined in a Philips 208 S EM at 80 kV.

### Cytotoxicity assay

Cell viability of CHO-K1 cells incubated with bacteria was determined by the lactate dehydrogenase (LDH) release assay as described by Bergmeyer and Bernt^58^, using the LDH Cytotoxicity assay kit (Innoprot, Spain). % Cytotoxicity = (Experimental—Blank)/Control—Blank) × 100.

### Preparation of ACT-coated beads

Coating of latex beads with purified ACT was performed by incubation of 10^6^ beads with 80 μg ACT or BSA in DMEM on a rocker for 1 h at room temperature. Quantification of the ACT bound to the beads was performed by western blot using purified ACT as standard. Approximately 5.1 μg ± 1.9 of ACT/10^6^ beads was determined. ACT or BSA-coated latex beads were washed three times in PBS, pH 7.4, then cells were incubated with the coated beads (Bead/CHO-K1 = 10) at 37 °C and 5% CO_2_ for 2 h, after which they were washed twice with DMEM, and processed for immunofluorescence analysis.

### Western blotting

Proteins were separated electrophoretically on 8.5% SDS-polyacrylamide gels and transferred to nitrocellulose membrane. The membranes were then blocked overnight at 4 °C, and after 2 h of incubation with the corresponding primary antibodies, membranes were washed and exposed to the secondary antibodies for 1 h at room temperature. Proteins were detected using the enhanced chemiluminiscence detection system (ECL®, Amersham Biosciences).

### Isolation of bacteria-containing endosomes from infected CHO-K1 cells

CHO-K1 cells were grown to 80% confluence in 175 T culture flask in DMEM as described above. Then, bacteria were added at a moi of 100 bacteria per cell. At different incubation times, medium containing free bacteria was discarded and cell monolayers were washed extensively with PBS at 37 °C to remove non-internalised bacteria. Then, bacteria containing-endosomes were isolated by discontinuous sucrose density gradient as described by Lürhmann and Haas^60^.

### Statistical Analysis

Significant differences between experimental groups were determined using Student´s *t* test. For all analysis a *p*-value of less than 0.01 was considered statistically significant.

## Additional Information

**How to cite this article**: Martín, C. *et al.* Adenylate Cyclase Toxin promotes bacterial internalisation into non phagocytic cells. *Sci. Rep.* **5**, 13774; doi: 10.1038/srep13774 (2015).

## Supplementary Material

Supplementary Information

## Figures and Tables

**Table 1:** Lipid composition of purified endosomal membranes purified after ACT-coated bacteria incubation with CHO-K1 cells.

	Cholesterol	Sphingomyelin	Phospholipids
% lipid composition	47.2 ± 3.4	31.8 ± 2.1	22.9 ± 7.5

Lipids were purified and quantified as previously described^61^.
