# Supplementary material for: Adenylate Cyclase Toxin promotes bacterial internalisation into non phagocytic cells
Source: Sci Rep. 2015 Sep 8;5:13774. doi: 10.1038/srep13774 (PMC4642564; doi:10.1038/srep13774)
Supplement: Supplementary Information [file srep13774-s1.pdf]

REVISED MANUSCRIPT

Manuscript Ref. SREP-15-02670A

Supplementary Information

Adenylate Cyclase Toxin promotes bacterial internalisation into non phagocytic cells

César Martín[a], Asier Etxaniz[a], Kepa B. Uribe[a], Aitor Etxebarria[a], David González-Bullón[a], Jon Arlucea[b], Félix M. Goñi[a], Juan Aréchaga[b] and Helena Ostolaza[a] *

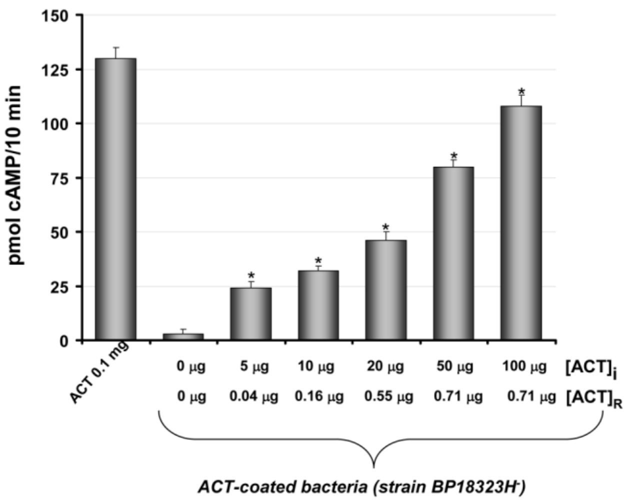

Supplementary Fig.S1

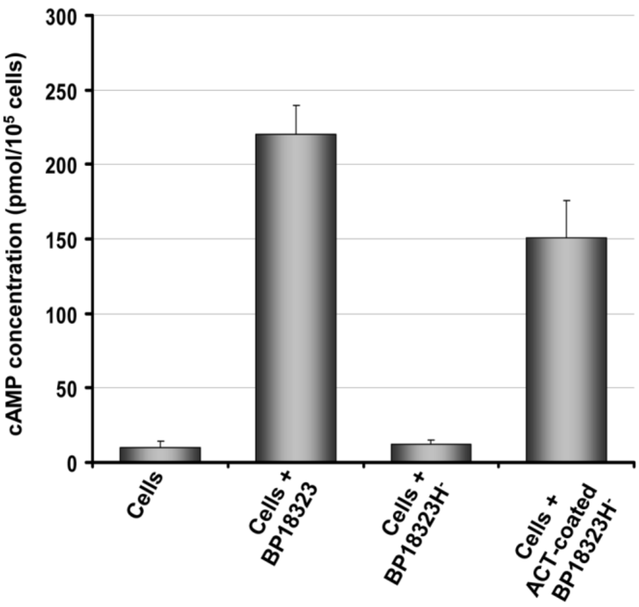

Supplementary Fig. S2

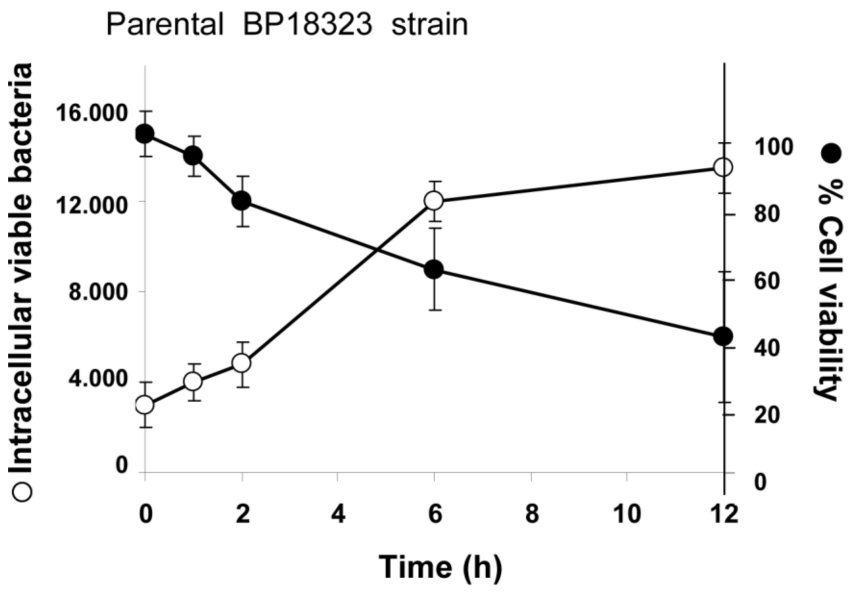

Supplementary Fig. S3

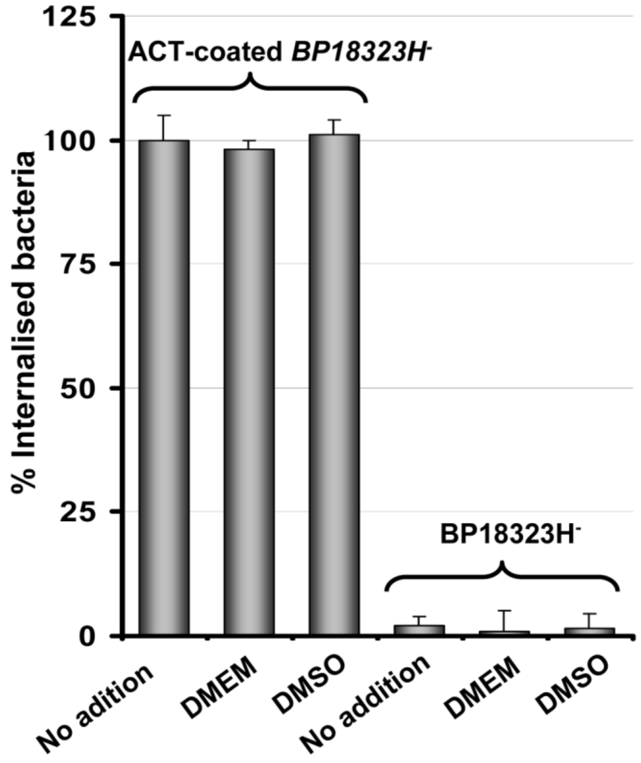

Supplementary Fig. S4

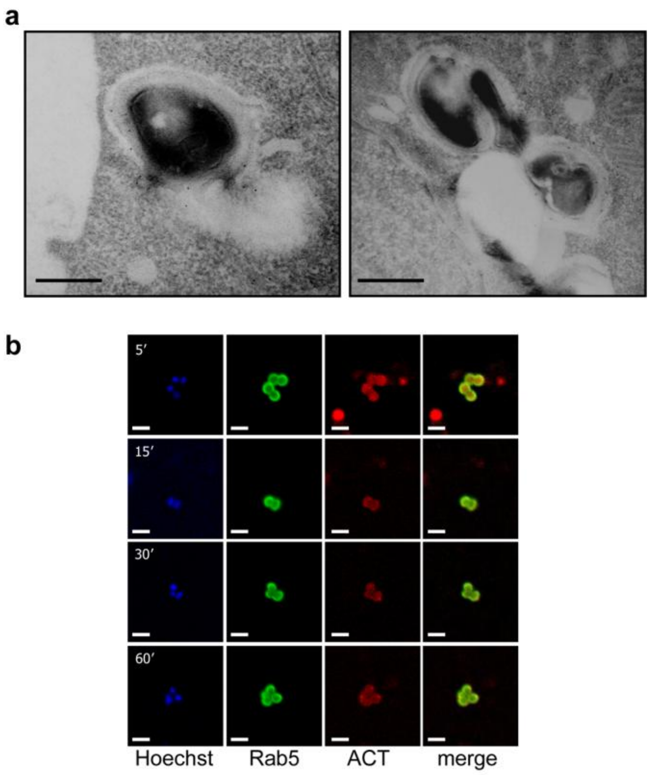

Supplementary Fig. S5

Supplementary Figure Legends

Supplementary Figure S1. Adenylate cyclase enzymatic activity of "ACT-coated bacteria". The adenylate cyclase activity *vs* the amount of toxin bound to non-virulent *B. pertussis* (strain BP18323H$^-$), was measured as described in *Methods* Section. We incubated bacteria (1 x 10^6 bateria) with different initial concentrations of pure ACT (0-100 μg/mL, $[ACT]_I$) for 60 min under constant stirring to favor contact. Bacteria were washed to eliminate unbound toxin and the "attached" ACT was quantified as described in *Methods* Section. Quantification of the real amount of ACT bound to the bacteria, $[ACT]_R$, was performed in three independent experiments and the obtained values ranged from 0.04 to 0.71 μg/mL μg of toxin bound per 10^6 bacteria. Data shown are the mean ± SD of at least three independent experiments.

Supplementary Figure S2. Quantification of the number of internalized bacteria. CHO-K1 cells were incubated with wt *B. pertussis*, with "ACT-coated bacteria" or with non-virulent strain BP18323$^-$ for 2 h and invasion was followed as described in *Methods* Section. The number of internalized bacteria was quantified as described in *Methods* Section. Data shown are the mean ± SD of at least three independent experiments.

Supplementary Figure S3. cAMP concentration in CHO-K1 cells incubated with wt *B. pertussis*, or "ACT-coated bacteria" or with uncoated strain BP18323H$^-$ for 2 h. Bacterial invasion was followed as described in *Methods* Section. cAMP concentration was quantified as described in *Methods* Section. Data shown are the mean ± SD of at least three independent experiments.

Supplementary Figure S4. Effect of vehicle solvents used in the characterization of the invasion pathway shown in Figure 4. CHO-K1 cells were pre-incubated for 30

min at 37 ºC with the vehicles in which the chemical inhibitors were dissolved. Then cell invasion was assayed as described in *Methods* Section. Chlorpromazine, sucrose and methyl-β-cyclodextrin were dissolved in DMEM, nystatin, genistein and okadaic acid, were dissolved in DMSO. The data were normalized to the control sample (*no addition*) and expressed as per cent of control entry. Data shown are the mean ± SD of at least three independent experiments performed in quintuplicate.

Figure S5. Endosomes purified from the infected cells were permeable to the dye DAPI. Confocal microscopy analysis of purified endosomes isolated from CHO-K1 cells infected with "ACT-coated bacteria" shows that bacteria-containing endosomes were permeable to the usually impermeable dye DAPI, which reached the endosome interior compartment and stained bacterial DNA. CHO-K1 cells were infected with "ACT-coated bacteria" and endosomes were purified and analysed as described in *Methods* Section, except that the permeabilization step with acetone was omitted.
